# Neuroprotective Effects of Ginseng Phytochemicals: Recent Perspectives

**DOI:** 10.3390/molecules24162939

**Published:** 2019-08-14

**Authors:** Xing Huang, Ning Li, Yiqiong Pu, Tong Zhang, Bing Wang

**Affiliations:** 1 School of Pharmacy, Shanghai University of Traditional Chinese Medicine, Shanghai 201203, China; 2 Experiment Center for Teaching and Learning, Shanghai University of Traditional Chinese Medicine, Shanghai 201203, China; 3 Institute of Chinese Materia Medica, Shanghai University of Traditional Chinese Medicine, Shanghai 201203, China; 4 Research Institute of KPC Pharmaceuticals, Inc., Kunming 650106, China; 5 Center for Pharmaceutics Research, Shanghai Institute of Materia Medica, Chinese Academy of Sciences, Shanghai 201203, China

**Keywords:** neuroprotective effects, ginseng, Alzheimer’s disease, ginsenoside, Parkinson’s disease

## Abstract

As our global population ages, the treatment of neurodegenerative diseases is critical to our society. In recent years, researchers have begun to study the role of biologically active chemicals from plants and herbs to gain new inspiration and develop new therapeutic drugs. Ginseng (*Panax ginseng* C.A. Mey.) is a famous Chinese herbal medicine with a variety of pharmacological activities. It has been used to treat various diseases since ancient times. Extensive research over the years has shown that ginseng has potential as a neuroprotective drug, and its neuroprotective effects can be used to treat and prevent neurological damage or pathologically related diseases (such as Alzheimer’s disease, Parkinson’s disease, Huntington’s disease, depression symptoms, and strokes). Moreover, evidence for the medicinal and health benefits of ginsenoside, its main active ingredient, in the prevention of neurodegenerative diseases is increasing, and current clinical results have not reported any serious adverse reactions to ginseng. Therefore, we briefly review the recent research and development on the beneficial effects and mechanisms of ginseng and its main active ingredient, ginsenoside, in the prevention and treatment of neurodegenerative diseases, hoping to provide some ideas for the discovery and identification of ginseng neuroprotection.

## 1. Introduction

As our global population ages, the treatment of neurodegenerative diseases is critical to our society. According to the World Health Organization, the percentage of the world’s population over the age of 60 will almost double from 12% in 2015 to 22% in 2050, reaching nearly 2 billion by 2050. In developed countries, the prevalence of known age-related neurodegenerative diseases such as Alzheimer’s disease (AD) [1] and Parkinson’s disease (PD) [2] has increased due to prolonged life expectancy. When it comes to AD, no one is a stranger. It has been more than 100 years since Dr. Alzheimer first described the disease in 1906. However, we have not found any effective means to prevent and treat this disease. Every year, hundreds of thousands of older adults suffer from this degenerative disease. These incurable diseases have devastating effects on patients and their families. Neurodegenerative diseases are mainly divided into chronic neurodegenerative diseases and acute neurodegenerative diseases. The former mainly includes AD, PD, Huntington’s disease, and the like. The latter includes mostly cerebrovascular accidents, stroke sequelae, and the like. Besides, depressive symptoms are often present in chronic conditions, as are chronic neurodegenerative disorders. Many patients with cognitive decline and dementia caused by AD and PD may have higher or lower levels of depressive symptoms at some point during their illness. Depressive symptoms are of particular importance in neurological diseases, especially in neurodegenerative diseases, because of the relationship between the brain, spirit, behavior, and mood [3]. The same is true of stress. The cause of neurodegenerative diseases as a group of heterogeneous diseases has not been fully elucidated. However, increased oxidative stress, protease resistance misfolding, chronic neuroinflammation, and the accumulation of aggregated proteins have been recognized as common mechanisms of most neurodegenerative diseases [4,5,6]. Recently, medicinal plants have received much focus, and disease-modifying drugs have been developed that can inhibit or delay the process of neurodegenerative processes [7]. As a well-known and popular Chinese medicine, ginseng has been found to have potential as a neuroprotective agent.

**Figure 1:**
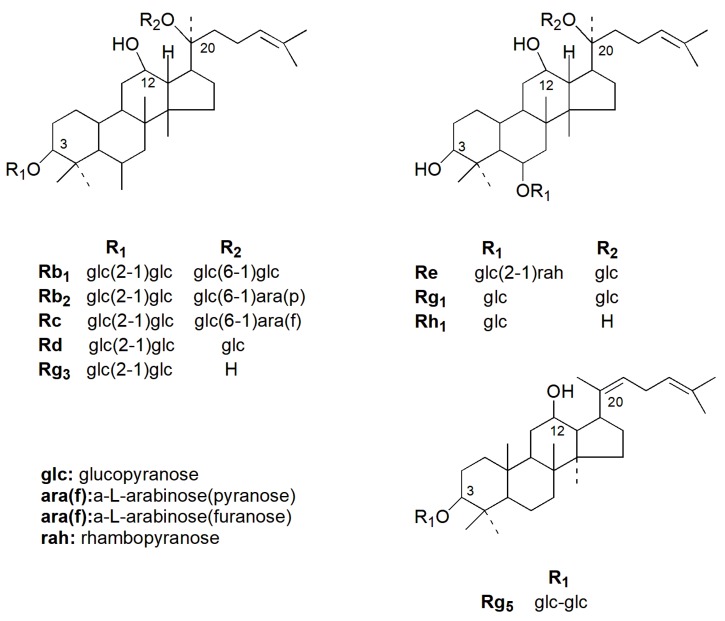
Structures of representative ginsenosides.

Ginseng is derived from the roots of *Panax ginseng* Meyer (Araliaceae) and has been used as a medication for thousands of years in East Asian countries, such as Japan, China, and Korea [8]. In China, ginseng has long been used as a drug whose clinical applications are extensive with proper development and utilization value. Ginseng promotes vitality, prolongs life, and shows therapeutic effects against various conditions, such as immune regulation, antitumor, antifatigue, antiaging, antioxidation, depression, diabetes, inflammation, dyspepsia, nervous system diseases, and many other aspects [9,10,11,12,13,14,15]. The various pharmacological activities of ginseng are attributed to its various active ingredients, such as ginsenosides, ginseng polysaccharides, volatile oils (terpenoids, alcohols, fatty acids, etc.), peptides, and amino acids [16,17]. One study showed that ginsenosides are the main active ingredient of ginseng responsible for its therapeutic and pharmacologic effects [18]. So far, more than 180 species of ginsenosides have been identified, according to reports [19,20]. Although all ginsenosides have different chemical structures, four-ring hydrophobic structures are familiar to them [21]. From a chemical viewpoint, these glycosides are classified into the 20(*S*)-protopanaxadiol type, which contains ginsenosides Rb1, Rc, Rb2, Rd, and Rg3, and the 20(*S*)-protopanaxatriol group, which includes ginsenosides Rg1, Re, Rg2, and Rh1 depend on different glycosides [22] (Figure 1). In fresh ginseng, Rb2, Rb1, Re, Rg1, and Rc are the main ginsenosides (70–80% of total ginsenosides) [23].

Ginsenosides may play a beneficial therapeutic role in some degenerative diseases, such as cardiovascular disease [24], glaucoma [25], cancer [26], and stroke [27,28,29]. As the main active ingredients of ginseng, 20(*S*)-protopanaxadiol and 20(*S*)-protopanaxatriol groups have gradually become the focus of medical and pharmaceutical research. These compounds also show advantageous pharmacological effects in the central nervous system (CNS), which can be converted therapeutically into clinical applications. Ginsenosides can strengthen brain function, prevent neuroinflammation and oxidative stress, and reduce or weaken a variety of neurodegenerative disorders, such as PD, AD, traumatic brain injury, and Huntington’s disease (HD) [30,31,32,33].

**Figure 2:**
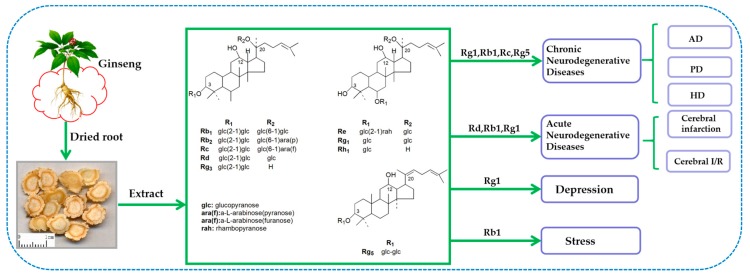
The review’s profiling for neuroprotective effects of ginseng phytochemicals.

Many reviews over the years have demonstrated the neuroprotective effects of ginseng and suggest that ginseng has potential as a therapeutic drug for various degenerative neurological diseases. However, they have extensively reviewed the treatment of ginseng in a certain degenerative nervous system disease [34,35,36] or covered ginseng treatment in multiple degenerative nervous systems but have not provided an in-depth review [37,38]. On the basis of these predecessors, this article comprehensively reviews the latest research and development of the beneficial effects and mechanisms of ginseng and its main active ingredient ginsenoside in the treatment and prevention of neurodegenerative diseases and hopes to discover and identify the neuroprotective effects of ginseng (Figure 2).

## 2. Chronic Neurodegenerative Diseases

Chronic neurodegenerative diseases are relatively common and difficult to treat, including AD, PD, and HD [39,40]. Neurodegeneration is a gradual loss of neuronal structure and function [40,41], including neuronal death and glial cell balance, leading to cognitive impairments such as dementia. Among other causes, age (AD, PD) and genetic mutations (HD, early-onset AD or PD, etc.) that affect the function of CNS cells cause chronic neurodegenerative diseases.

Degenerative diseases in the CNS have exhibited an increased incidence rate and have gradually occurred in young people. A large number of in vivo and in vitro experiment research results have shown that ginseng has significant neuroprotective activity. Neuroprotection can be defined as a therapeutic intervention (that is, to prevent neuronal death) to reduce disease progression and to delay overprogression from preclinical to clinical by delaying or blocking the neurodegenerative process or delaying the purpose of neuronal death [42]. At present, the neuroprotective effect of ginseng has been increasingly recognized and investigated. The following is a summary of evidence for the role of ginsenosides in some degenerative diseases and their clinical significance.

### 2.1. Alzheimer’s Disease

As the most common type of dementia, AD is known as a neurodegenerative disease caused by the degenerative disorders of the CNS [1]. Epidemiological studies have shown that AD mainly occurs in the elderly over 65 years old, after which its incidence increases with age [43]. The main pathological feature of AD is extracellular β-amyloid (Aβ) plaques and intracellular neurofibrillary tangles in the brain. There are also neuronal and white matter losses and inflammatory responses in the relevant brain regions. A significant disability associated with AD is cognitive impairment [44], which interferes with work, activities, relationships, and leisure in daily life. The incidence of AD has increased and has become a substantial economic burden on patients’ families and society due to the large aging population. Although AD is a relatively common and age-related severe disease, its etiology and exact pathogenesis remain unclear, and few treatments are available to reduce or prevent brain cell deterioration [45]. Therefore, it is essential to develop potential therapeutic drugs to avoid or delay AD-related memory decline and to study their effects on the brain of AD patients [46]. These processes, in which multiple etiological and pathological factors work together to modulate the ultimate pathophysiology of the disease, are essential in AD [47,48]. Ginsenosides are the main pharmacologically active ingredients in ginseng: they have been reported to have various effects on the CNS and have become more and more popular as a means of improving cognitive ability in recent years [49].

Rg1 is a representative monomer in ginseng. Modern pharmacological studies have shown that Rg1 can act on the nervous system in the treatment of elderly AD. However, Rg1 is easily degraded by the intestinal bacteria after oral administration: the bioavailability is only 1% to 20% and is quickly eliminated in the blood [50]. Therefore, the parenteral route might be a potential alternative. The well-characterized transgenic AD mice over expressing amyloid precursor protein/Aβ and nontransgenic littermates at age of six and nine months were treated with Rg1 for three months via intraperitoneal injection (i.p.) [51]. They assessed changes in amyloidosis, neuropathology, and behavior in mice. The results found that Rg1 had significant and multifaced neuroprotective effects in their AD mouse model. They noted that Rg1 improved amyloid pathology, modulated the amyloid precursor protein process, improved cognition, and activated hippocampal-dependent protein kinase/hippocampal-respond element-binding protein (PKA/CREB) signaling to induce neuroprotection. These findings show the potential of Rg1 for new classes of drugs for AD treatment and provide new ideas for its treatment.

Chen et al. [52] found that compared to oral Rg1 physiological saline solution, a nasal administration route increased Rg1 distribution and transport efficiency by 5.05 times and 2.50 times, respectively, and shortened the time to 100% transport in the brain (nasal spray compared to Rg1 saline in the nasal cavity). The agent increased Rg1 distribution and transport efficiency by 11.80 times and 3.35 times, respectively, and the area under the curve in the brain increased. Therefore, for the application of Rg1 for the prevention and treatment of neurodegenerative diseases, nasal administration is a good route of administration, and nasal spray is an excellent dosage form.

However, the manner of nasal administration is highly irritating, and the patient’s compliance is low. On this basis, no obvious irritating transdermal preparation is the right choice. He et al. [53] used a Franz diffusion pool and HPLC on Rg1 in an in vitro transdermal study, and the results showed that Rg1 could penetrate rat abdominal skin without any penetration agent. Lu Jia et al. [54] evaluated the permeability of Rg1 by studying the transdermal absorption of Rg1 in injured sites in vitro. The results showed that Rg1 could penetrate rat abdominal skin and that the steady-state permeation rate *J* = 0.161 mg·cm^−2^·h^−1^ with a time lag of 0.368 h. Sun Yuan et al. [55] investigated the transdermal permeation of Rg1 in cataplasm. The permeation rate *J* = 40.63 μg·cm^−2^·h^−1^ was determined by high performance liquid chromatograph, and the cumulative permeation amount at 24 h was 173.075 mg·cm^−2^. They showed that Rg1 had skin permeability, which can prolong medication time, reduce the number of times, and improve the patient’s medication compliance when prepared in a transdermal absorbent dosage form.

Rb1 is a kind of ethanol-soluble compound from ginseng with a high abundance in total ginsenosides, with an excellent safety record. Studies have shown that Rb1 achieves neuroprotection by combating various neurotoxins. A recent study used Aβ amyloid damage primary cultured hippocampal neurons to establish an AD cell model to explore the protective effect and mechanism of ginsenoside Rb1 on hippocampal neurons induced by Aβ amyloid it was found that Rb1 showed neuroprotective effects in three ways, namely (1) promoting neural growth, (2) promoting the expression of growth-promoting kinases and helping to prevent their levels from decreasing, and (3) playing the role of an antiapoptotic agent after Aβ-induced apoptosis [56].

Rb1 has been shown to protect the brain from the toxicity caused by aluminum. Zhao et al. [57] treated mice with drinking water containing AlCl_3_ (200 mg·kg^−1^ bodyweight) for six months, followed by a post-treatment of Rb1 oral administration (20 mg·kg^−1^ per day) for the next four months. They found that treatment with Rb1 obviously improved memory and learning and reduced tau phosphorylation by reversing the glycogen synthase kinase 3β and the protein phosphatase level. The results indicated that Rb1 protected mice from Al-induced toxicity. The mechanism of action may be to prevent tau hyperphosphorylation by modulating glycogen synthase kinase 3β and protein phosphatase levels, suggesting that Rb1 is a potential prophylactic drug for AD and other neurodegenerative diseases associated with tau pathology.

### 2.2. Parkinson’s Disease

PD is also one of the most common types of neurodegenerative disease, ranking only after AD [58]. The main pathological change in PD is the chronic degeneration of dopaminergic neurons in the substantia nigra pars compacta (SNpc), resulting in decreased dopamine content in the striatum (caudate and putamen) [59,60]. The main symptoms of PD are dyskinesia, including tremors, stiffness, movement retardation, sleep disorders, cognitive impairment, autonomic dysfunction, and depression [61]. These symptoms lead to degenerative changes in the dopaminergic pathway of the SN and striatum. The current treatment methods are mainly divided into two types: one is a symptomatic treatment for motor symptoms, and the other modifies potential diseases by protecting or restoring neurons. However, the current pharmacological treatment for PD remains at the symptomatic treatment stage, there has been no further development, and this symptomatic treatment has no way to lose dopaminergic neurons in PD patients progressively. Thus, it is vital to have an in-depth understanding of the molecular mechanism of PD and to discover new PD therapeutic agents with high efficiency [62].

The protective effect of ginsenoside extracted from ginseng on the toxicity of α-synuclein has been evaluated. Of all the ginsenosides (i.e., Rb1, Rg3, and Rg1) screened, only Rb1 was shown to be a potent inhibitor of alpha-synuclein fibrillation and toxicity, thereby acting as a defibrillation. Therefore, Rb1 can be used as a drug for treating PD and related diseases [63]. When exposed to a toxic compound (methyl-polypyridine-iodide) mouse dopaminergic cells began to die, and neurite length was shortened. In addition, the level of immune-positive tyrosine hydroxylase cells decreased. Rb1 and Rg1 were administered to counteract this process. Although these compounds could not prevent cell loss, they prevented neuronal degeneration by increasing the length of neurites in surviving neurons, thereby suggesting their partial neuroprotective effect [64].

Ginsenoside Rg1 is a vital chemical component of ginseng, with low toxicity, neuroprotective, and anti-inflammatory effects. A recent study investigated the potential of Rg1 to treat PD and used in vivo and in vitro models of PD to investigate whether Rg1 exerts neuroprotection through the Wnt/β-catenin signaling pathway. In an in vivo study of this experiment, dopaminergic cell loss induced by 1-methyl-4-phenyl-1,2,3,6-tetrahydropyridine (MPTP) in a dose-dependent manner was reduced after models of PD rats were administered an i.p. injection of Rg1 for 15 consecutive days. In vitro, after pretreatment with Rg1, cell viability was enhanced, and 1-methyl-4-phenylpyridinium (MPP+)-induced cell apoptosis was reduced. The results demonstrated that Rg1 shows neuroprotective effects not only in in vivo but also in in vitro PD models, and these effects act through the Wnt/β-catenin signaling pathway [62]. Another study found that MPTP-induced animal survival rates, the loss of dopamine neurons, motor deficits, and abnormal ultrastructural changes in the SNpc were significantly improved by oral Rg1 administration. In addition, they also found that the anti-neuroinflammatory properties of Rg1 may be involved in neuroprotection. In their experiments, Rg1 was found to exhibit neuroprotective effects in a chronic MPTP/propionate-induced PD mouse model. Increased blood brain barrier (BBB) permeability caused by inflammation in the CNS may lead to immune cells and peripheral toxins entering the brain and thus causing degeneration, which is characteristic of PD. In the SNpc and striatum of MPTP-treated mice, an infiltration of CD4+ and CD8+ T lymphocytes could be found [65].

Zhou et al. [66] confirmed that i.p. administration of Rg1 for 15 consecutive days could protect anti-tyrosine hydroxylase positive cells in the SNpc region from MPTP toxicity measured with immunofluorescence and found that CD3+ T cells accumulated in the SNpc and that Rg1 treatment reduced the infiltration of T cells in this region. They demonstrated the immunoprotection of Rg1 in an MPTP-induced PD mouse model and provided a new therapeutic approach for the treatment of PD from an immunological perspective. Table 1 summarizes the basic and clinical evidence for the beneficial effects of ginseng on AD and PD in a more intuitive way.

### 2.3. Huntington’s Disease

HD is an autosomal-dominant, inherited, progressive neurodegenerative disorder caused by the repeated expansion of cytosine–adenine–guanine in the Huntington gene on chromosome 4 [74], in which the patients exhibit numerous symptoms, including cognitive, motor, and behavioral impairments, in which clinical heterogeneity during the disease is common [75,76,77]. This dysfunction contributes to the expression of the clinical symptoms of HD. Up to now, there has been no drug treatment available on the market to improve or prevent HD effectively. Since there are no available treatments to assess progressive neuronal dysfunction, behavioral and psychiatric symptoms can only be managed through the use of conventional therapies.

Considerable efforts have been recently devoted to the use of herb and botanical-derived products to prevent and treat HD. To evaluate the neuroprotective activity of ginsenosides, Wu et al. [78] investigated the effect of 10 different ginsenosides on HD by using an in vitro model of the mouse medium-sized multispinous striatum nerve YAC128. Ginsenosides exhibited a protective effect on 200 µM glutamate-induced apoptosis after pretreatment with saponins Rb1, Rc, and Rg5. However, the seven other samples of ginsenosides (Re, Rd, Rg3, Re, Rh1, Rd, a combination of Rg5 and Rk1, and a mixture of Rk3 and Rh4) had no protective effect on the glutamate-induced apoptosis of multispinous striatum nerve YAC128 cells. At a concentration of 1 µM, Re, Rh1, and a mixture of Rk1 and Rg5 exhibited toxicity. Subsequent studies have shown that the neuroprotective effects of Rb1, Rc, and Rg5 are associated with an inhibition of glutamate-induced changes in Ca^2+^ concentrations. These results indicate that Rb1, Rg5, and Rc may serve as a medicine for the treatment of neurodegenerative diseases, including HD.

The exact cause of neuronal cell death in HD is unknown. Systemic administration of 3-nitropropionic acid (3-NP), which is an irreversible succinate dehydrogenase inhibitor, induces cellular adenosine triphosphate depletion and causes a selective striatal degeneration similar to that observed in HD. A previous study evaluated the in vitro and in vivo effects of ginsenosides on striatal neurotoxicity induced by 3-NP repeat treatment in rats. They dissolved ginsenosides in saline and administered different doses to rats i.p.. The results suggested that 3-NP-induced rat striatum degeneration is inhibited by ginsenosides. These compounds were shown to increase the survival rate of HD animals, inhibit intracellular Ca^2+^ elevation, and reduce behavioral disorders after toxin administration [79].

## 3. Acute Neurodegenerative Diseases

Acute neurodegenerative diseases mainly include cerebrovascular accidents, stroke sequelae, and the like. A stroke is a brain obstruction or rupture in the brain’s blood supply caused by disease, with high mortality and high disability, and it is the leading cause of elderly permanent disability and death. The incidence of stroke in developed countries is lower than in developing countries, and stroke is a more serious problem in developing countries [80]. Stroke is mainly divided into two types: one is an ischemic stroke, primarily due to infarction, and the other is a hemorrhagic stroke, mainly due to intracerebral/parenchymal or subarachnoid hemorrhage. These two types of strokes affect the brain in different ways. Ischemic strokes and hemorrhagic strokes account for 85% and 15% of strokes, respectively: about 6 million people die each year from stroke, and the lifetime risk of stroke is estimated to be 8% to 10% [81,82]. Currently, the most effective way to treat cerebral infarction is to restore the blood supply by recanalization of the occluded arteries or by endovascular therapy or thrombolytic agents immediately after arterial occlusion. However, recanalization therapy may make brain damage worse: this is called ischemia-reperfusion injury (I/R), and it results in poor clinical outcomes because of fatal edema (brain herniation) or intracranial hemorrhage after thrombolysis [83]. Thrombolytic therapy is effective for acute ischemia, but its application is limited by time windows and contraindications. Currently, there is no effective treatment for strokes caused by neuronal damage and death [84].

### 3.1. Cerebral Infarction

Ginsenosides have various pharmacological effects and limited clinical efficacy in the treatment of stroke and other cerebrovascular diseases. Among the different neuroprotective drugs currently used, Rd ginsenosides have apparent clinical efficacy on acute cerebral ischemia [85,86,87]. To date, studies have shown that Rb1 exhibits neuroprotection in a rodent ischemic model. Some researchers have investigated the effects of Rb1 on early and delayed brain injuries in a nonhuman primate thromboembolic stroke model [88]. They showed that by injecting autologous blood clots into the left internal carotid artery, occlusion of the middle cerebral artery could induce a thromboembolic stroke. Rb1 at 300 μg·kg^−1^ per day or saline was administered into the cephalic vein of the forearm for seven days before embolization on the day following embolization. Subsequently, a positive reaction was evaluated by several measurements, and it was shown that Rb1 did improve early and delayed damage in a thromboembolic stroke model of nonhuman primates.

One of the main causes of brain damage is bleeding, leading to cerebral edema. Recent trials have shown that Rb1 plays an important role in improving complications following an ischemic brain event. In that study [89], a rat model of subarachnoid hemorrhage was used, and Rb1 was intravenously administration at a dose of 20 mg·kg^−1^ 30 min after the first brain injury caused by subarachnoid bleeding. The same treatment was continued for seven days. Then, brain edema significantly reduced, and neurobehavioral functioning improved. In addition, a histological examination showed a significant reduction in lumen thickness and basilar artery vasospasms with treatment. The experimental results showed that Rb1 could resist brain damage caused by hemorrhage.

To assess the therapeutic and preventive effects of Rb1 on a neural injury during cerebral infarction, Jiang et al. used a model of middle cerebral artery occlusion (MCAO) in rats to study the effects of Rb1 with edaravone (a neuroprotective agent) as a control. Rb1 was intragastrically administered to the rats either before or after MCAO surgery to determine its therapeutic and preventive effects. The infarct volume of rats treated with Rb1 was less than that of edaravone, which suggests the potential of ginsenosides as a neuroprotectant [90]. Studies have shown that ginsenosides promote ischemia and brain damage after the proliferation and differentiation effects of neural stem cells (NSCs). Hypoxia-inducible factor-1α (HIF-1α) is a transcription factor in hypoxia for the proliferation of and differentiation of NSCs that plays an important regulatory role of vascular endothelial growth factor and HIF-1α in hypoxia in the expression of cytokines are closely related to nerves and blood vessels. In a microenvironment under the condition of cerebral ischemia and hypoxia, ginsenosides regulated the proliferation and differentiation of NSCs through the HIF-1α–vascular endothelial growth factor (VEGF) pathway. Ginsenosides acting on the expression of HIF-1α in the nest were controlled by the expression of HIF-1α, and the expression of VEGF in the downstream target gene was promoted. The paracrine mode helped the cascade of microvascular proliferation and neurogenesis, regulated NSCs, and promoted the proliferation and differentiation of NSCs. It lays the foundation for the transplantation of NSCs and treatment of stroke and other brain damage to the CNS diseases.

Studies have shown that ginsenosides can promote coculture conditions in the proliferation of NSCs and induce NSCs to differentiate neurons and glial cells in the case of cerebral ischemia and hypoxia. This mechanism may be related to the control of ginsenosides in the HIF-1α–VEGF pathway. Ginsenosides can act on the astrocytes in the nest. Brain injury repair and brain function restoration can be achieved by regulating the transcription of the HIF-1α gene, promoting the activation of HIF-1α, initiating the expression of VEGF downstream of the target gene, acting in a paracrine manner in NSCs, improving the microenvironment of NSCs, promoting the proliferation and, differentiation of NSCs to play a neuroprotective effect [91]. Zheng et al. [84] conducted an experiment on male Wistar rats. Three days before the establishment of the permanent MCAO model, the rats were intraperitoneally injected with 25-mg·kg^−1^·d^−1^ doses of total ginsenoside or the same volume of normal saline. The same treatment was continued until the animals were killed on the 1st, 3rd, 7th, and 14th day. Neurological function was blindly assessed. The results demonstrated that ginseng total saponins could improve neurological function deficits after focal cerebral ischemia by enhancing adult CNS regeneration and inducing the endogenous activation of NSCs. In a previous study, participants were selected using a replacement zonal randomization method based on the National Institutes of Health Stroke Scale score and randomized into two treatment groups. Patients simultaneously received a placebo or ginsenosides at a dose of 1 mg·kg^−1^ per body (maximum 100 mg) with 10% recombinant tissue plasminogen activator as a bolus followed by the remaining 90% as a constant infusion for 60 min. This supported the clinical use of ginsenosides as a potential supplement with recombinant tissue plasminogen activator treatment, which can reduce symptomatic intracerebral hemorrhage and improve the treatment outcome of stroke patients [92].

### 3.2. Cerebral Ischemia-Reperfusion Injury

Cerebral ischemia-reperfusion injury (I/R) is a complex pathophysiological event that is associated with hypoxia and the energy depletion of brain tissue during ischemia, ultimately leading to neuronal destruction. High recurrence rates, high mortality, and high disability are the resulting characteristics of the damage and cascade reactions caused by cerebral ischemia. However, so far, only a few antithrombotic drugs, such as heparin, recombinant tissue plasminogen activator, and aspirin [93], can be used for the treatment of ischemic stroke, and the safety issues associated with reperfusion injury and hemorrhage are uncontrollable. Therefore, several potential neuroprotective agents should be investigated for the treatment of cerebral ischemia and reperfusion injury to reduce safety concerns caused by antithrombotic drugs in ischemic stroke.

The four compounds Rb1, Rg1, schizandra, and DT-13 ((25(*R*,*S*)-ruscogenin-1-*O*-[β-d-glucopyr-anosyl-(1→2)][β-d-xylopyranosyl-(1→3)]-β-d-fucopyra-noside)) combined in a specific ratio (6:9:5:4 *w*/*w*, called SMXZF) showed neuroprotective activity. In the experiments of Guo et al. [94], male C57BL/6 mice were subjected to I/R injury using right MCAO for 1 h with subsequent 24-h reperfusion. Three doses of SMXZF (4.5, 9, and 18 mg·kg^−1^) were i.p. administered after ischemia for 1 h. An autophagic inhibitor, 3-methyladenine (300 µg·kg^−1^), was i.p. administered 20 min before ischemia as a positive drug. The results demonstrated that SMXZF showed neuroprotection through three pathways. First, SMXZF inhibited autophagy via a reduction in autophagosomes. Second, SMXZF increased cerebral blood flow and counteracted infarction. Finally, SMXZF significantly inhibited the phosphorylation of adenosine monophosphate-activated protein kinase and the mammalian target of rapamycin and the expression of c-Jun N-terminal kinase and its phosphorylation induced by 24-h reperfusion. Their study revealed that SMXZF displayed neuroprotective effects against focal I/R. In another study, cerebral ischemia was evaluated by a 2-h occlusion of the middle cerebral artery and reperfusion followed by i.p. administration of Rb1 (40 mg·kg^−1^) and saline. The result demonstrated that Rb1 promoted neural behavior recovery, increased brain-derived neurotrophic factor expression, decreased caspase-3 (an essential component of an apoptotic pathway) activity, and induced neurogenesis after cerebral ischemia. These results provided new evidence for the protection of neurons by Rb1from reperfusion injury [95].

Rg1 has been proven to possess protective effects over the CNS. In a study by Wang Rui et al. [96], rats were administered with Rg1 (45 mg·kg^−1^) by intravenous injection (i.v.) 1 h before focal ischemia and 3 h after reperfusion. The results showed that Rg1 exhibited a potent neuroprotective effect by significantly reducing neurological scores and the brain infarct volume in MCAO rat models. The active ingredient of ginseng, Rg1, was shown to scavenge free radicals and improve antioxidant capacity. The pathogenesis of neurons injured by cerebral ischemia is complicated and is associated with oxygen-free radical injury, inflammatory factor damage, excitatory amino acid injury, and intracellular Ca^2+^ overload [97]. Among these injuries, oxidative stress-induced oxygen-free extreme trauma has attracted considerable attention [98]. Brain tissues contain abundant unsaturated fatty acids and are susceptible to damage by free radicals [99]. The results of Sun et al. [100] indicated that Rg1 resisted oxidative stress and free radical injury, increased the survival rate of damaged cells, reduced the amount of leaked lactate dehydrogenase and caspase-3 activation, increased superoxide dismutase (SOD) activity and heat shock protein 70 expression, and suppressed cell apoptosis in a dose-dependent manner. Table 2 lists some basic and clinical evidence for the beneficial effects of ginseng on I/R.

These previous studies have shown that Rg1 exerts significant neuroprotective effects and is neurotrophic in hypoxic ischemic insults in vivo and in vitro [103]. On the basis of these studies, several researchers have built a focal cerebral ischemia model that is induced by MCAO. The researchers conducted i.p. administration of Rg1 on rats at a dose of 40 mg·kg^−1^·d. They used Longa’s five-point scale to perform neurological examinations, the brain infarct volume was determined by 2, 3, 5-triphenyltetrazolium chloride staining, and the permeability of the BBB was evaluated by Evans blue dye. Western blot and quantitative reverse transcription-polymerase chain reaction were used to assess protease-activated receptor-1 expression. The results indicated that Rg1 alleviates neurological dysfunction and reduces the BBB permeability in I/R rats. The mechanisms by which Rg1 prevents focal cerebral ischemia includes the regulation of protease-activated receptor-1 expression [102].

Rd is another active ingredient in ginseng. Much clinical research has proven that the treatment of acute ischemic stroke with Rd is effective and safe [11]. Considerable studies have shown that Rd protects neurons from I/R by decreasing redox injury, inhibiting apoptosis, and maintaining mitochondrial function [103,104,105,106]. Ye et al. [107] used Rd with concentrations ranging from 0.1 mg·kg^−1^ to 200 mg·kg^−1^ or vehicle applied i.p. 30 min before MCAO and found that when the dose of Rd was 10–50 mg·kg^−1^, the infarct volume was significantly reduced and improved long-term neurological outcomes up to six weeks after ischemia. This results demonstrated the neuroprotection of Rd in transient focal ischemia, which may involve an integrated process of scavenging pathways of early free radicals and a late anti-inflammatory effect. Ye et al. [108] evaluated the protective role of Rd in attenuating ischemic neuronal injury through cell experiments. In addition, Rd stabilized the mitochondrial membrane potential and attenuated the apoptotic death of hippocampal neurons after oxygen–glucose deprivation exposure. From these findings, it can be seen that Rd has potential as a neuroprotective agent for cerebral ischemic injury, and further research is meaningful and worthy of encouragement. Rd reduced the BBB permeability and alleviated the neurological dysfunction in I/R rats. The mechanisms by which Rd prevents focal cerebral ischemia include the regulation of protease-activated receptor-1 expression [102]. Xie et al. [101] administered Rd (50 mg/kg) or an equal volume of saline containing 10% 1,3-propanediol (*v*/*v*) by i.p. 30 min before MCAO surgery or immediately after an MCAO operation. The results showed that in a MCAO rat model and a neuron model of oxygen–glucose deprivation culture, Rd significantly improved the infarct volume, behavioral score, and viability of cultured neurons after I/R. Rd decreased the overexpression of the *N*-methyl-d-aspartate receptor 2B subunit in the membrane and its hyperphosphorylation after I/R. Rd protected Sprague Dawley rats and cultured neurons from I/R by inhibiting the overexpression of the *N*-methyl-d-aspartate receptor 2B subunit in the membrane and its hyperphosphorylation. This indicates that Rd is a potentially useful drug for preventing or treating I/R.

## 4. Depression

Depression, which has become a prevalent psychiatric disorder, has been shown to be associated with notable changes in specific brain regions [109]. The main symptoms of depression are depression, lack of pleasure and mental retardation. It is a chronic, recurrent episode of emotional psychosis, and patients have a serious suicidal tendency. The prevalence of depression has increased annually because people face various pressures, and the pathogenesis of the study has attracted considerable attention [110]. The pathogenesis of depression is unclear because the medical community mainly uses two categories, namely monoamine neurotransmitter theory and non-monoamine neurotransmitter pathogenesis (which includes non-monoamine neurotransmitters, a focus of study in recent years). At present, treatments for depression are mainly western medicine treatments, psychological treatments, and hospital care therapy. A large number of clinical applications and experimental studies have found that traditional Chinese medicine, especially ginseng, plays a role in antidepression, and its antidepressant effects have attracted the attention of many researchers.

Gap junctional dysfunction between astrocytes in the prefrontal cortex is associated with major depressive disorder. In one study, rats were exposed to chronic unpredictable stress and were administered Rg1 (5, 10, and 20 mg·kg^−1^) for 28 days intragastrically, and a forced swimming test and sucrose preference were used to analyze depressive symptoms in rats [111]. Their findings demonstrated that Rg1 remarkably alleviated depression-like behavior in rats. This research provided a new perspective on the use of Rg1 as an antidepressant and opened up the possibility of studying the natural bioactive substances used in traditional Chinese medicines, which may have high efficiency and few side effects, to treat depression.

Some studies have found that oral ginseng extract causes a therapeutic effect on women with menopausal depression. Jeong et al. [112] randomly selected 35 women aged 18–65 years from outpatients, administered red ginseng, and used the Montgomery–Asberg Depression Scale and Depression Symptom Scale to assess depressive symptoms. The severity of depression significantly improved, which indicated that Korean red ginseng as an adjuvant therapy could effectively change the residual symptoms of patients with depression. Subsequently, Lee et al. [113] expanded the clinical sample size by randomly dividing 93 postmenopausal women into two groups: the first group was administered a placebo, and the second group was administered ginseng capsules. After two weeks, a blood test and a depression scale questionnaire were given to the two groups. Depressive symptoms were relieved in patients with depression in the ginseng group.

## 5. Stress

Stress can decrease the levels of a brain-derived neurotrophic factor (BDNF) and make change in heat shock protein-70 which are valuable as anti-stress agents to induce dysfunction in the brain part known as hippocampus. Rb1 administration increases the levels of heat shock protein-70 and BDNF, eliminates the effects of stress, and avoids any damage to the hippocampus [114]. Ginsenosides have been recognized as neuroprotective agents, including playing a role as antistress ingredients. The main active compounds of ginseng, including the ginsenosides Rb1 and Rg3, were administered to stress gerbil mice, and the levels of stress marker polyamines, especially putrescine levels, were evaluated. In the experimental results, Rb1 and Rg3 obviously reduced the levels of stress markers, acted against stress, and established themselves as stress control elements [115,116].

Mice exposed to stress were treated with Rb1 and assessed for stress-induced levels of brain monoamine. The results indicated that Rb1 reversed stress-induced changes in brain monoamine levels, and Rb1 was an antistress factor [117]. Tyrosine kinase B (TrkB), whose concentrations may play an essential role in neurosurvival and differentiation, is an important member of the kinase family and is a neurotrophic factor. Immobilized stressed rats were treated with Rb1, and it was found that Rb1 increased TrkB levels by upregulating mRNA. At the same time, stress-relieving levels of adrenocorticotropic hormone and corticosterone (CORT) increased. Therefore, Rb1 actively resisted immobilization stress [118]. Ginsenosides have been demonstrated to be antistress agents in previous studies. In one study, ginsenosides were found to be associated with learning ability and sleep deprivation stress. Sprague Dawley rats were exposed to sleep pressure to affect their learning ability and were then subjected to Rb1 treatment. Later experiments showed that Rb1 obviously increased the level of somatostatin, which is a neuromodulator. Furthermore, learning and memory skills improved [119]. A number of studies have focused on assessing the role of Rb1 as a neuroprotective agent by improving mitochondrial stability. After Rb1 treatment, SH-SY5Y cells were subjected to glucose and oxygen stress. Rb1 suppressed toxic oxygen radicals and increased mitochondrial membrane stability to significantly increase cell viability. Rb1 also promoted Bcl-2, which is a potent antiapoptotic factor, and counteracted increasing levels of proapoptotic Bax. Therefore, Rb1 could increase neuronal cell protection and mitochondrial stability [120].

## 6. Conclusions and Future Perspectives

Over the years, evidence for the medicinal and health benefits of ginseng in the prevention of neurodegenerative diseases has increased, and there are currently no clinically reported severe adverse reactions. Therefore, the unique ability of ginseng to prevent neurodegeneration is increasingly of concern to consumers and researchers. Most experiments on the treatment of neurodegeneration are aimed at determining the effects of drugs on neurons, but neglecting the support and nutrition of neuromuscular fibers in neuronal growth. Tests have shown that ginseng can be used as a drug to affect neurons [121] and to achieve peripheral nerve regeneration to prevent muscle atrophy.

Ginseng and its saponins are useful as drugs for the treatment of brain diseases caused by their neuroprotective effects. At present, there are two main aspects to this. One is the relationship between the prototype of ginsenoside and its activity, and the other is the study of its metabolic profile and the association of metabolites with activity. Research on the transformation of flora in the digestive tract has been relatively detailed, but metabolism research in the body is still difficult. This is the main direction that should be addressed in future research on ginseng and its saponins.

The beneficial effects of ginseng and its biologically active ingredients, ginsenosides, have been reported and have attracted considerable attention from researchers. Most previous studies have been conducted in cellular and animal models and have focused on neurons. Most ginsenosides had lower bioavailability and a less popular distribution in the brain. The transport capacity of ginsenosides through the BBB was relatively limited because they were not detected in the brain tissue of MCAO and normal rats. Although their transport through a BBB-damaged model (in vitro), which was induced by hydrogen peroxide, was slightly enhanced, the results in that study indicated that the permeability of the BBB was poor. The low permeability of the BBB is the primary obstacle to overcome in for the treatment of brain diseases. Although varying degrees of brain disease will be associated with local BBB damage, but the formation of BBB low permeability of the key structure of the existence of close connections still limit the drugs into the brain to achieve effective diagnosis and treatment concentration. Therefore, a highly efficient and safe method should be developed for the treatment of CNS diseases. A study on the delivery of drugs across the BBB has provided new ideas for drug delivery strategies in the brain and guidance for the construction of nanomedicine in the diagnosis and treatment of brain diseases such as PD and AD. At present, the domestic dosage of Chinese patent medicines containing ginsenoside is enormous: some are oral, and there are many traditional Chinese medicine injections, all of which are aimed at this type of disease. Therefore, clinical practice in China is prosperous, but there is a lack of systematic research integration and reassessments of post-marketing efficacy, and especially a lack of senior clinical research. Future researchers should conduct research integration and re-evaluations of post-marketing efficiency, particularly to provide high-level theoretical studies of clinical research and thoroughly elucidate cellular and molecular mechanisms and provide a number of signal transduction pathways to reveal the mystery of ginsenosides.

In summary, previous studies on ginsenosides have powerfully shown that ginsenosides and their metabolites/derivatives are potential stocks as active agents in the prevention and treatment of brain diseases in the future. In addition, their neuroprotective effects and mechanisms of action are worthy of further research and proof by scholars.

## Figures and Tables

**Table 1:** Summary of the treatment of ginsenosides in Parkinson’s disease (PD) and Alzheimer’s disease (AD).

Ginsenoside	Activities	Models	Dosing and Administration	Mode of Action	Ref.
20(*S*)-proto-panaxadiol nanocrystals	Neurodegenerative disease	A polyethylene catheter was cannulated into the right jugular vein of the rats under anesthesia	20 mg/kg Oral	Increased plasma Cmax and increased bioavailability	[67]
Korean red ginseng extract	PD	The rats were administered 1-methyl-4-phenyl-1,2,3,6-tetrahydropyridine (MPTP)–HCl by i.p.	100 mg/kg Oral	Restored MPTP-induced protein downregulation	[61]
Rg1	PD	The rats were administered MPTP–HCl by i.p.	5mg/kg, 10mg/kg, and 20mg/kg i.p.	Decreased MPP+-induced cytotoxicity Protected PC12 cells from MPP+-induced apoptosis.	[62]
Rg1	PD	The chronic MPTP/probenecid model	10 mg/kg, 20 mg/kg, or 40 mg/kg Oral	Improved high MPTP-induced behavior defects, loss of dopamine neurons, mortality, and abnormal ultrastructure changes in the SNpc	[65]
Rg1	PD	Two weeks after ovariectomy, unilateral infusion of lipopolysaccharide into the right side of substantia nigra pars compacta (SNpc) under anesthesia	Rg1 10 mg/kg, 10 mg/mL i.p.	Showed protective effects on mesencephalic dopaminergic neurons	[68]
Panax ginseng extract	PD	Injected a solution of rotenone in the right striatum of rat bregma	100 mg/kg Oral	Improved the midbrain and striatal changes and showed a partial ameliorative effect against a rat model of PD	[69]
Rb1	AD	SH-SY5Y cells used stable isotope labeling with amino acids in cell culture	100 mM/day Rb1 pretreatment	Prevented β-amyloid-induced neurotoxicity in SH-SY5Y cells and apoptotic cells; increased the expression of actin cytoskeleton proteins	[70,71,72]
Ginseng total saponins	AD	SAM, senescence-accelerated mouse; SAMP, senescence-accelerated mouse prone substrain; SAMR, senescence-accelerated mouse-resistant substrain	50,100, and 200 mg/kg/day Oral	Prevented memory loss in aged SAMP8 mice by upregulating the increase in antioxidant capacity in the hippocampus and upregulating plasticity-related proteins	[73]

**Table 2:** The ginsenoside experimental method for treating I/R.

Ginsenoside	Models	Dosing/Administration	Mode of Action	Ref.
Rd	Induced by transient MCAO	50 mg·kg^−1^ i.p.	Improved behavior score, viability, and infarct volume of the cultured neurons after ischemia and protected Sprague Dawley rats and cultured neurons from I/R.	[101]
Ginseng total saponins	Induced by transient MCAO	25mg·kg^−1^·d^−1^ i.p.	Improved the regeneration of the central nervous system in adults, thereby improving neurological deficits after focal cerebral ischemia.	[84]
Rg1	Induced by transient MCAO	45 mg·kg^−1^ i.v.	Showed effective neuroprotection by reducing the brain infarct volume and neurological scores.	[96]
Rg1	Induced by transient MCAO	40 mg·kg^−1^·d^−1^ i.p.	Showed it was neuroprotective by improving neurological damage, BBB permeability, and the brain infarct volume.	[102]

MCAO: middle cerebral artery occlusion; BBB: blood–brain barrier.

## Abbreviations

AD, Alzheimer’s disease; Aβ, amyloid beta; BBB, blood–brain barrier; CNS, central nervous system; HD, Huntington’s disease; HIF-1α, hypoxia-inducible factor-1α; i.p., intraperitoneal injection; i.v., intravenous injection; I/R, ischemia-reperfusion injury; MCAO, middle cerebral artery occlusion; MPP+, 1-methyl-4-phenylpyridinium; MPTP, 1-methyl-4-phenyl-1,2,3,6-tetrahydropyridine; 3-NP, 3-nitropropionic acid; NSCs, neural stem cells; PD, Parkinson’s disease; Rb1, ginsenoside Rb1; Rd, ginsenosides Rd; Rg1, ginsenoside Rg1; SN, substantia nigra; SNpc, substantia nigra pars compacta; SOD, superoxide dismutase; VEGF, vascular endothelial growth factor; PKA/CREB, hippocampal-dependent protein kinase/hippocampal-respond element-binding protein.
